# Enhanced Recovery and Bioactivity of Flavonoids From Peanut Shells (*Arachis hypogaea*): Antioxidant and Acetylcholinesterase Inhibitory Properties

**DOI:** 10.1002/fsn3.70457

**Published:** 2025-06-19

**Authors:** Tuyet Nhung Tran, Quang Tien Ho, Nhat Tan Nguyen, Thao Vy Nguyen, Anh Vo Bui, Van‐Son Dang, Dieu‐Hien Truong, Hoang Chinh Nguyen, Colin J. Barrow

**Affiliations:** ^1^ Faculty of Applied Sciences Ton Duc Thang University Ho Chi Minh City Vietnam; ^2^ Institute of Tropical Biology Vietnam Academy Science and Technology Ho Chi Minh City Vietnam; ^3^ Division of Endocrinology, Diabetes and Metabolism The University of Alabama at Birmingham Birmingham Alabama USA; ^4^ Centre for Sustainable Bioproducts Deakin University Geelong Victoria Australia; ^5^ College of Health Sciences Abu Dhabi University Abu Dhabi UAE

**Keywords:** acetylesterase inhibition, antioxidant activity, flavonoids, fractionation, peanut shells, quercetin

## Abstract

Peanut shells, a by‐product of the peanut processing industry, are rich in flavonoid compounds with a range of bioactivities. This study aimed to develop an efficient extraction and fractionation process to enhance the recovery and in vitro antioxidant and acetylcholinesterase (AChE) inhibition activities of these compounds from peanut shells cultivated in Vietnam. Peanut shell samples were subjected to maceration with ethanol, followed by liquid–liquid partitioning (LLP) and column chromatography (CC) using a hexane and ethyl acetate solvent system at varying ratios. This process yielded a crude ethanolic extract (CEE) and its fractions: chloroform (CHF), petroleum ether (PEF), ethyl acetate (EAF), acetone (ACF), and eluted fractions (F1–F4). The developed extraction and fractionation significantly enhanced the total flavonoid content (TFC), from 65.49 mg QE/g in CEE to 759.80 mg QE/g in fraction F2, and quercetin content from 13.46 μg/g (CEE) to 292.38 μg/g (fraction F2). The CEE and its fractions were evaluated for antioxidant activity using a DPPH radical scavenging and AChE inhibitory activity. A strong positive correlation was observed between the TFC and both bioactivities, with activity ranked as follows: F2 > EAF > F3 > F1 > F4 > CHF > PEF > CEE > ACF. Among these samples, fraction F2 demonstrated the highest bioactivities, with IC_50_ values of 16.00 μg/mL for DPPH scavenging and 47.22 μg/mL for AChE inhibition. These findings suggest that the developed systematic extraction and fractionation process, employing maceration, LLP, and gradient elution CC, is a promising method for the efficient isolation of flavonoid‐enriched fractions from peanut shells, with the resulting fractions exhibiting both antioxidant and AChE inhibitory activities.

## Graphical abstract

Peanut shells, a by‐product of the peanut processing industry, are rich in flavonoid compounds with notable bioactivity. This study aimed to develop an efficient extraction and fractionation process to enhance the recovery and bioactivities of these compounds from peanut shells cultivated in Vietnam. An efficient process was established for enhancing flavonoid content from peanut shells and these compounds showed antioxidant and AChE inhibitory activity.

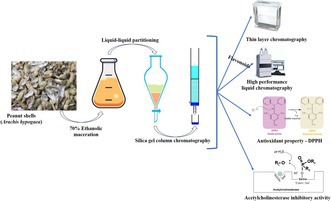

## 1 Introduction

Peanuts (groundnut, *Arachis hypogaea* , family Leguminosae) are among the most significant food crops in tropical and subtropical regions worldwide, particularly in Asia, Africa, and America. Vietnam has emerged as a leading exporter of peanut‐derived products (Çiftçi and Suna 2022; Kim et al. 2023). Peanuts are globally cultivated for their seeds and oil (Bertioli et al. 2011; Adhikari et al. 2019). Before processing into products like peanut oil, butter, or roasted kernels, the outer yellow, woody shells (referred to as peanut hulls) are removed (Kim et al. 2023; Kiene et al. 2023). These shells, accounting for 230–300 g/kg of harvested peanuts, represent an abundant and low‐cost byproduct (Adhikari et al. 2019; Kiene et al. 2023). Despite being considered waste, peanut shells are a rich source of bioactive compounds safe for human consumption, including polyphenols, flavonoids, luteolin, carotene, and isosaponaretin (Yu et al. 2014; Adhikari et al. 2019; Hassan et al. 2022; Imran et al. 2022). Phenolic content ranges from 428.1–739.8 μg gallic acid equivalents per gram (mg GAE/g), total flavonoid content (TFC) from 142.6 to 568.0 μg quercetin equivalents per gram (μg QE/g), and luteolin content from 0.25 to 1.12 mg per gram (Adhikari et al. 2019; Imran et al. 2022). Compounds such as quercetin, luteolin, and eriodictyol have shown therapeutic potential against Alzheimer's disease and diabetes mellitus (Kou et al. 2022; Zhu et al. 2024), highlighting their potential as functional ingredients.

Although peanut shells are predominantly discarded or repurposed as livestock feed, plastic fillers, and fuel feedstock (Dean 2020; Imran et al. 2022; Kim et al. 2023), their rich phenolic and flavonoid profiles confer antioxidant, anti‐diabetic, antibacterial, and anti‐enzymatic activities (Demirtas et al. 2013; Adhikari et al. 2019; Wang et al. 2021; Dean 2020; Imran et al. 2022; Çiftçi and Suna 2022). Studies have demonstrated strong antioxidants and antimicrobial activities from peanut shell extracts (Bodoira et al. 2022) and significant acetylcholinesterase (AChE) inhibition from related plant extracts containing similar flavonoids (Yaman et al. 2024). However, the specific role of peanut shells as AChE inhibitors, a mechanism critical for Alzheimer's disease therapy, remains underexplored. Given their abundant phenolic content and bioactivities, further investigation into peanut shells' therapeutic potential is warranted.

Peanut shell composition varies by maturity, variety, and processing methods (Hoang et al. 2008; Liao et al. 2021; Sorita et al. 2023). Boiling and roasting, for example, enhance TFC and antioxidant activity, with roasted shells showing superior DPPH radical and H_2_O_2_ scavenging capacities (Hassan et al. 2022). Extraction techniques also play an important role. Imran et al. (2022) demonstrated that ultrasound‐assisted ethanolic extraction achieved higher polyphenol recovery in just 10 min compared to conventional methods requiring an hour. This emphasizes the importance of optimizing extraction parameters to maximize the yield and bioactivity of flavonoid‐enriched extracts. Different techniques and solvents can produce extracts with varying flavonoid levels, leading to differing biological effects (Munhoz et al. 2014; Kaczorová et al. 2021). Dean (2020) provided a comprehensive review of extraction procedures, ranging from simple single‐solvent methods to advanced separation techniques for isolating and identifying polyphenolic compounds from peanut skins, highlighting their diverse applications in food and medicine.

To address these knowledge gaps, this study investigated the effect of extraction and fractionation techniques on the recovery of flavonoids and bioactive properties from peanut shells cultivated in Vietnam. The peanut shell samples were macerated with ethanol, and the resulting ethanolic extract was sequentially fractionated using liquid–liquid partitioning and silica gel column chromatography, followed by analysis with advanced chromatographic techniques. The crude extract and its fractions were then assessed for antioxidant properties and AChE inhibition. This investigation also aimed to establish peanut shells as a sustainable resource for high‐value bioactive compounds, offering both health benefits and a solution to agricultural waste management challenges.

## 2 Materials and Methods

### 2.1 Chemicals

The following chemicals and reagents were obtained from Sigma‐Aldrich (Singapore): sodium nitrite (NaNO_2_), sodium hydroxide (NaOH), ferric chloride (FeCl_3_), aluminum chloride (AlCl_3_), acetylthiocholine iodide (ATCh), 5,5′‐dithio‐bis‐[2‐nitrobenzoic acid] (DTNB), 2,2‐diphenyl‐1‐picrylhydrazyl (DPPH), AChE from electric eel (type VI‐S, 349 U/mg solid, 411 mg/U protein), silica gel 60, ascorbic acid (vitamin C), and berberine chloride. The analytical grade solvents such as ethanol (EtOH), methanol (MeOH), petroleum ether (PE), ethyl acetate (EtAc), hexane (Hex), acetone (Me_2_CO) chloroform (CHCl_3_), and toluene were also purchased from Sigma‐Aldrich (Singapore). Phosphate‐buffered saline (PBS) and Tris–HCl were obtained from Thermo Fisher Scientific (Singapore).

### 2.2 Crude Extract Preparation

Peanut fruits ( *Arachis hypogaea* ) were obtained from Mo Duc, Quang Ngai, Vietnam (March 2024) and authenticated by the Institute of Tropical Biology, Vietnam. The peanut fruits were manually deshelled, and the shells were dried at 60°C before being ground using a grinder (ZXMOTO, China). The ethanolic crude extract (CEE) from peanut shells was obtained using the method described by Liu et al. (2022), with minor modifications. Briefly, the powdered shells were macerated in 70% aqueous ethanol (1:10, w/v) for 24 h. The mixture was incubated in a shaking incubator (60°C, 150 rpm) for 4 h, centrifuged at 10,000 × g for 10 min at room temperature, and filtered through a vacuum filtration system (Rocker, Taiwan). This extraction was repeated thrice to ensure maximum recovery of bioactive compounds, and the combined supernatants were concentrated using a rotary evaporator (Pollabm, India) at 60°C to yield CEE.

### 2.3 Liquid–Liquid Partitioning

The CEE of peanut shells was subjected to liquid–liquid partitioning, following methods described in previous studies, with minor modification (Idowu et al. 2016; Nguyen et al. 2024). In particular, the CEE was reconstituted in distilled water (1:10, w/v) and partitioned sequentially using PE, CHCl_3_, EtOAc, and Me_2_CO (1:1, v/v) using a separating funnel. The resulting fractions—PEF, CHF, EAF, and ACF—were concentrated using a vacuum rotary evaporator, air‐dried, freeze‐dried, and stored in the dark at 4°C for further analysis.

### 2.4 Determination of Flavonoid Compounds Using Silica Gel Column Chromatography

The flavonoid‐rich fraction from CEE was further separated using silica gel column chromatography (CC). A column (240 cm × 20 cm) was packed with silica gel slurry (150 g silica gel 60, 230–400 μm, 350 mL hexane). This procedure was adapted from previous studies with minor modifications (Lin et al. 2016; Pham et al. 2023). The fraction was loaded onto the column after being adsorbed onto silica gel 60 powder in ethanol and dried. Elution was performed using hexane:ethyl acetate mixtures (90:10, 70:30, 50:50, and 20:80; v/v) at a flow rate of 30 drops/min. Fractions (30 mL) were collected, concentrated using rotary vacuum evaporation, and labeled as F1–F4.

### 2.5 Determination of Extraction Yield and TFC

The extraction yield (EY) was calculated as follows (Truong et al. 2019):

(1) $$\mathrm{EY}\;\left(\%\right)=\frac{W_{1}}{W_{2}}\times 100$$

where *W*_1_ is the weight of the extract/fraction obtained and *W*_2_ is the weight of the sample used for extraction/fractionation.

The TFC was measured using the aluminum chloride colorimetric assay, as described by Nguyen et al. (2024). Briefly, distilled water (4 mL) was added to 1 mL of sample (1 mg/mL), followed by 0.3 mL of 5% NaNO_2_. After 5 min of incubation, 0.3 mL of AlCl_3_ solution was added, and the mixture was incubated for 6 min. Subsequently, 2 mL of NaOH and 2.4 mL of distilled water were added, and the mixture was incubated for 10 min. The absorbance of the resulting solution was measured at 510 nm. The TFC was quantified as mg QE/g based on a calibration curve (*y* = 0.0017*x* – 0.0018, *R* ^2^ = 0.9952).

### 2.6 Thin Layer Chromatography

Thin layer chromatography (TLC) was used to identify quercetin and other flavonoid compounds in the CEE and its fractions as described by Nguyen et al. (2024) with minor modifications. Quercetin standard and samples (1 mg/mL in 70% EtOH) and were spotted onto silica gel 60 F_254_ plates, developed in a mobile phase (toluene:EtOAc:formic acid, 7:3:1 v/v/v), dried at 105°C for 5 min to evaporate any residual solvent, and visualized under UV light (254 nm and 365 nm) using a UV Viewing Cabinet (UVP 95‐0072‐06, Analytik Jena, USA) after spraying with 5% FeCl_3_ in EtOH. Retention factors (*R*_f_) were calculated using the formula described by Charoensumran et al. (2021):

(2) $$R_{f}=\frac{\text{Distance traveled}\;\mathrm{by}\;\text{the compound}}{\text{Distance traveled}\;\mathrm{by}\;\text{the solvent}}$$

### 2.7 High Performance Liquid Chromatography Analysis

High performance liquid chromatography (HPLC) analysis was carried out using an Agilent 2160 system equipped with Eclipse Plus C18 column (4.6 × 250 mm, 5 μm), based on the method described by Garg (2021) with minor modifications. Samples (1 mg/mL) were prepared by dissolving them in the mobile phase, a MeOH:water mixture (7:3, v/v). The analysis were performed at a detection wavelength of 360 nm, with an injection volume of 20 μL and a mobile phase flow rate of 1 mL/min. Quercetin in the samples was identified based on its retention time (RT) and quantified using a calibration curve generated with a quercetin standard (*y* = 0.0008*x* − 0.0017, *R* ^2^ = 0.9955), where *x* denotes quercetin concentration and *y* represents the peak area.

### 2.8 Determination of DPPH Radical Scavenging Activity

The antioxidant activity of peanut shell samples was evaluated using the DPPH free radical scavenging assay, based on the method reported by Adhikari et al. (2019) with slight changes. A freshly prepared DPPH solution (0.004% in MeOH) was used as the reagent. One milliliter of samples (20–100 μg/mL) was incubated with 1 mL of DPPH solution (0.004% in MeOH) for 30 min at 37°C. The absorbance of each reaction solution was measured at 517 nm using a UV–Vis spectrophotometer (V‐730, Jasco, USA). The scavenging activity was calculated as follows:

(3) $$\text{DPPH radical scavenging activity}\;\left(\%\right)=\frac{A_{\text{control}}-A_{\text{sample}}}{A_{\text{control}}}\times 100$$

where *A*_control_ is the absorbance of DPPH + MeOH and *A*_sample_ is the absorbance of DPPH + sample. Ascorbic acid was employed as the positive control. The IC_50_ value (the concentration required to inhibit 50% of DPPH radicals) was determined by plotting the percentage inhibition against the extract concentrations.

### 2.9 AChE Inhibition Assay

The in vitro AChE inhibitory activity of the peanut shell extracts was determined using Ellman's colorimetric method, as modified by Eldeen et al. (2005), with ATCh as the substrate. The reaction mixture containing 200 μL of extract (50–200 μg/mL), 20 μL of AChE (2 U/mL), and 80 μL of DTNB (50 mM Tris–HCl, pH 7.7, with 0.02 M MgCl_2_·6H_2_O and 0.1 M NaCl) was pre‐incubated for 5 min at 25°C. The reaction was then initiated by adding 15 μL of ATCh (14 mM). After 5 min of incubation, the absorbance was measured at 405 nm. Berberine chloride (2–8 μg/mL in phosphate buffer) served as the positive control. The AChE inhibition was calculated as follows:

(4) $$\text{Inhibition}\;\left(\%\right)=100-\frac{A_{t}}{A_{c}}\times 100$$

where *A*_*t*_ is the absorbance of the sample (extract) and *A*_*c*_ is the absorbance of the control. The IC_50_ value was determined by plotting the percentage inhibition against extract concentration.

### 2.10 Statistical Analysis

All experiments were conducted in triplicate, with data expressed as mean ± standard deviation (SD). Statistical significance (*p* < 0.05) was evaluated using Tukey's multiple‐comparison test in Minitab 20 software (State College, Pennsylvania, USA).

Multivariate analyses, including principal component analysis (PCA) and heatmap with clustering analyses, were performed to evaluate clustering patterns among extracts and fractions. Square Euclidean distances were employed to calculate variables between the peanut shells crude extract and its fractions. Principal components (PCs) were validated through full cross‐validation at a 95% confidence level, with variables standardized as *Z* scores (mean = 0, SD = 1). For clustering variables, the complete linkage method was applied, and the resulting dendrogram's variable scale ranged from 0 (highest variable) to 0.45 (lowest variables).

## 3 Results

### 3.1 Effect of Fractionation on Total Flavonoid and Quercetin Content

Flavonoids, a diverse class of naturally occurring phenylchromones, are ubiquitously found in various plant materials. In this study, the extraction yield and TFC of CEE and its fractions derived from peanut shells were determined (Table 1). The extraction with 70% ethanol yielded 21.08% CEE. The CEE was then fractionated, producing 10.88% ACF, 10.33% PEF, 6.08% CHF, and 5.18% EAF. Among these fractions, EAF showed the highest TFC and was selected for a further fractionation, yielding 49.76% fraction F4, 24.60% fraction F3, 16.51% fraction F2, and 9.40% fraction F1.

**TABLE 1:** Extraction yield, total flavonoid, and quercetin contents of different fractions obtained peanut shells.

Sample	Extraction yield (%, w/w)	TFC (mg QE/g)	Quercetin (μg/g)
CEE	21.08 ± 0.16^c^	65.49 ± 2.38^h^	13.46 ± 0.36^e^
PEF	10.33 ± 0.74^e^	118.82 ± 2.12^g^	—
CHF	6.08 ± 0.05^g^	131.37 ± 1.48^f^	—
EAF	5.18 ± 0.07^h^	357.45 ± 3.92^b^	74.42 ± 0.43^b^
ACF	10.88 ± 0.36^e^	26.08 ± 2.90^i^	—
F1	9.40 ± 0.34^f^	205.69 ± 1.23^d^	16.34 ± 0.87^d^
F2	16.51 ± 0.54^d^	759.80 ± 4.4^a^	292.38 ± 0.54^a^
F3	24.60 ± 1.15^b^	301.37 ± 1.70^c^	24.56 ± 0.59^c^
F4	49.76 ± 2.25^a^	194.76 ± 2.05^e^	14.63 ± 0.41^e^

*Note:* Data are presented as mean ± SD (*n* = 3). Values with different superscript lowercase letters within a column indicate statistically significant differences (*p* < 0.05). F1–F4, eluted fractions obtained from mixture of hexane:ethyl acetate at ratios of 90:10, 70:30, 50:50, and 20:80 (v/v), respectively. The extraction yield of CEE was calculated based on the weight of peanut shell used for extraction. The yields of PEF, CHF, EAF, and ACF were calculated based on the weight of CEE, while the yields of fractions F1–F4 were calculated based on the weight of EAF.

Abbreviations: ACF, acetone fraction; CEE, crude ethanolic extract; CHF, chloroform fraction; EAF, ethyl acetate fraction; PEF, petroleum ether fraction.

Among the obtained extracts from peanut shells, the highest TFC was observed in fraction F2 (759.80 mg QE/g), followed by EAF, F1, F4, CHF, PEF, and CEE with TFC ranging from 65.49 mg QE/g to 357.45 mg QE/g. Similarly, the highest quercetin content was recorded in fraction F2 (292.38 μg/g), followed by EAF (74.42 μg/g), F3 (24.56 μg/g), F1 (16.34 μg/g), F4 (14.63 μg/g), and CEE (13.46 μg/g). No quercetin was detected in PEF, CHF, and ACF.

### 3.2 TLC and HPLC Characterization

**FIGURE 1:**
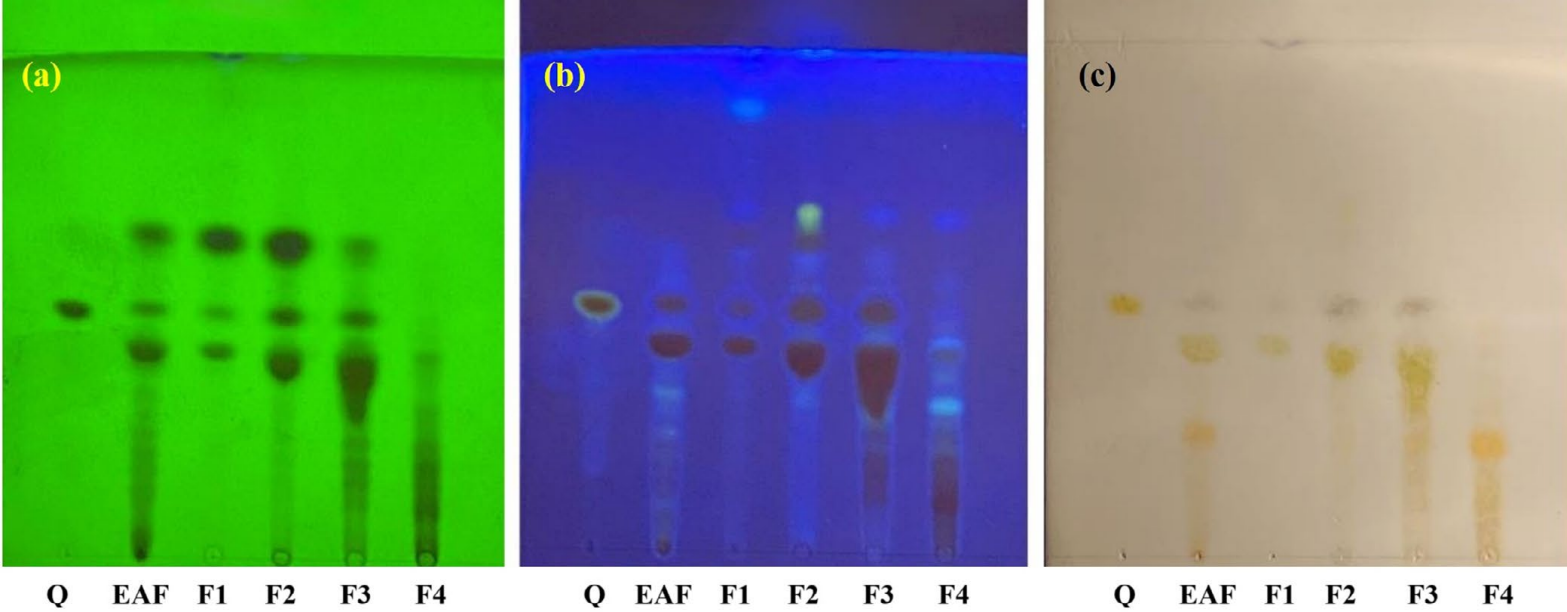
TLC profiling of the peanut shell extract and fractions: (a) spots at UV 254 nm, (b) spots at UV 365 nm, and (c) spots after FeCl_3_/EtOH reagent treatment. EAF, ethyl acetate fraction; F1–F4: Fractions eluted using hexane:ethyl acetate at ratios of 90:10, 70:30, 50:50, and 20:80 (v/v), respectively; Q, quercetin standard.

The TLC profiles of the EAF and fractions F1–F4 from peanut shells are presented in Figure 1. Fluorescent spots were analyzed under UV light at 254 and 366 nm using a UV Viewing Cabinet. At 254 nm, the EAF and fractions F1–F4 exhibited 3, 3, 3, 4, and 4 distinct spots, respectively, while under 366 nm, these fractions displayed 4, 5, 6, 5, and 4 spots, respectively. Bioactive compound screening via TLC, particularly for quercetin, is summarized in Table 2. All fractions showed the presence of quercetin with an *R*_f_ value of 0.44, corresponding to the standard. Additionally, flavonoids were detected in all samples, as indicated by yellow or purple spots on the TLC plate after treatment with FeCl_3_/EtOH reagent (Figure 1c).

**TABLE 2:** *R*_f_ values and the number of spots observed at UV wavelengths 254 and 356 nm for the ethyl acetate fraction (EAF) and its eluted fractions (F1–F4) from peanut shells.

No.	Wavelength UV_254_					Wavelength UV_365_				
	EAF	F1	F2	F3	F4	EAF	F1	F2	F3	F4
1	—	—	—	—	—	—	0.89	—	—	—
2	—	—	—	—	—	—	0.64	0.64	—	—
3	0.59	0.59	0.59	0.59	—	—	0.59	0.59	—	—
4	—	—	—	—	—	—	—	0.56	0.56	0.56
5	0.44	0.44	0.44	0.44	0.44	0.44	0.44	0.44	0.44	0.44
6	0.36	0.36	0.36	0.36	0.36	0.36	0.36	0.36	0.36	0.36
7	—	—	—	—	—	—	—	—	0.29	—
8	—	—	—	0.27	0.27	0.27	—	0.27	—	—
9	—	—	—		0.19	0.19	—	—	—	0.19
10	—	—	—				—	—	0.13	0.13
Total no. of spots	3	3		4	4	4	5	6	5	5

*Note:* Standard quercetin with an *R*_f_ value of 0.44. “—”: not detected.

**FIGURE 2:**
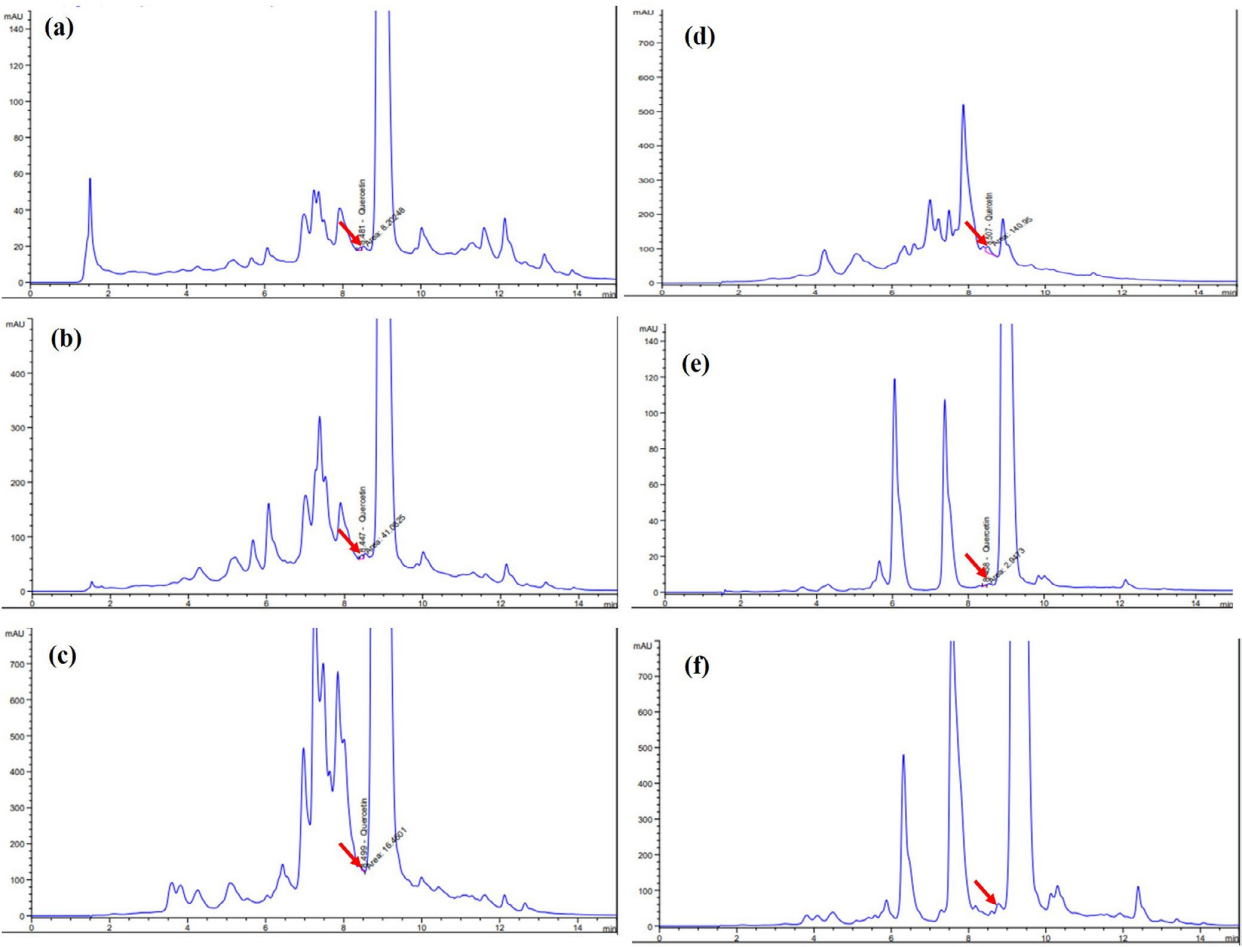
HPLC chromatograms of (a) CEE, (b) EAF, (c) F1, (d) F2, (e) F3, and (f) F4. Quercetin peaks are marked with arrows.

Figure 2 illustrates the HPLC‐UV chromatograms of the CEE, EAF, and fractions F1–F4. The quercetin peak, identified at a RT of 8.45 min, confirmed its presence across all the samples. HPLC quantification revealed significant enrichment of flavonoids, particularly quercetin, among the fractions (Table 1). In addition to quercetin, the HPLC analysis displayed the presence of other compounds, including a prominent peak at a RT of 9.25 min. While the identity of this compound remains undetermined, comparison with commercial flavonoid standards or further analysis using LC–MS is required to provide precise identification and validation.

### 3.3 DPPH Radical Scavenging Activity

**FIGURE 3:**
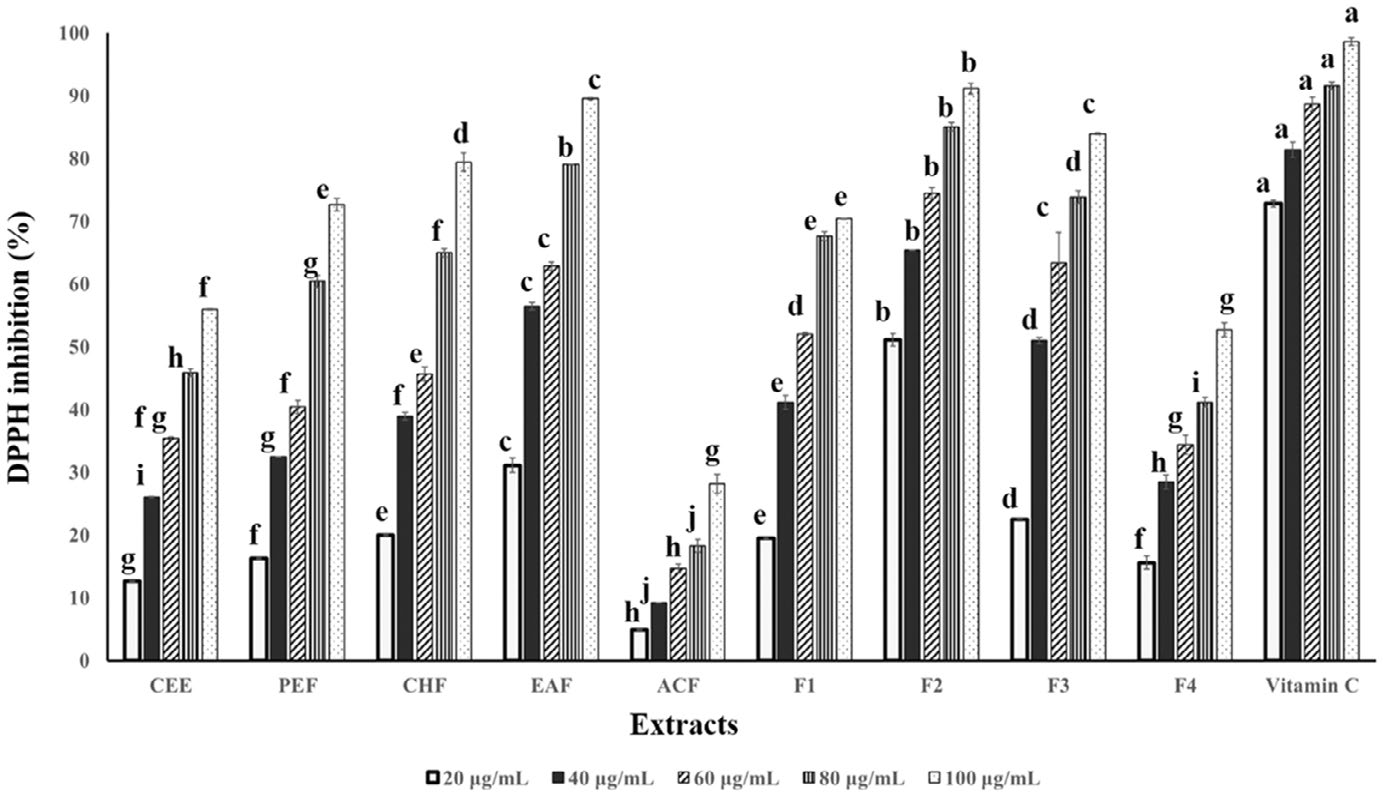
DPPH radical scavenging of peanut shell extracts. Data are shown as mean ± SD (*n* = 3). Means within the same color (concentration) with different letters significantly differ by Tukey's test at *p* < 0.05.

The antioxidant potential of peanut shell samples was evaluated using the DPPH radical scavenging assay (Figure 3). All samples demonstrated a dose‐dependent increase in percentage inhibition, indicating their ability to neutralize free radicals. At 100 μg/mL, the scavenging activity of the CEE was 55.92%, which significantly increased to 72.64%, 79.42%, and 89.52% following liquid–liquid partitioning with petroleum ether, chloroform, and ethyl acetate, respectively. The scavenging activity further improved with column chromatography. Based on the IC_50_ values of the tested extracts, where a lower IC_50_ indicates stronger activity, fraction F2 exhibited the highest scavenging activity, with an IC_50_ of 16.00 μg/mL, comparable to that of vitamin C (IC_50_ = 13.54 μg/mL), as shown in Table 3. Overall, the free radical scavenging activity of the samples followed the order: vitamin C > F2 > EAF > F3 > CHF > PEF > F1 > CEE > F4 > ACF.

**TABLE 3:** IC_50_ values for DPPH radical scavenging activity and AChE inhibitory activity of the peanut shell extracts.

Extracts	IC_50_ value (μg/mL)	
	DPPH test	AChE inhibition
CEE	87.63 ± 0.14^b^	246.82 ± 1.41^a^
PEF	68.12 ± 0.85^c^	232.08 ± 1.08^b^
CHF	65.13 ± 5.46^c^	160.22 ± 0.70^c^
EAF	33.79 ± 1.13^e^	107.56 ± 0.70^g^
ACF	> 100	> 250
F1	55.71 ± 0.35^d^	148.63 ± 2.00^d^
F2	16.00 ± 0.97^f^	47.22 ± 0.13^h^
F3	36.85 ± 1.55^e^	133.64 ± 0.47^f^
F4	94.50 ± 0.56^a^	140.30 ± 0.11^e^
Vitamin C	13.54 ± 1.00^e^	—
Berberine choline	—	4.92 ± 0.02^i^

*Note:* Data are shown as mean ± SD (*n* = 3). Values with different superscript lowercase letters within a column of each extraction factor indicate statistically significant differences (*p* < 0.05).

### 3.4 In Vitro AChE Inhibitory Activity

**FIGURE 4:**
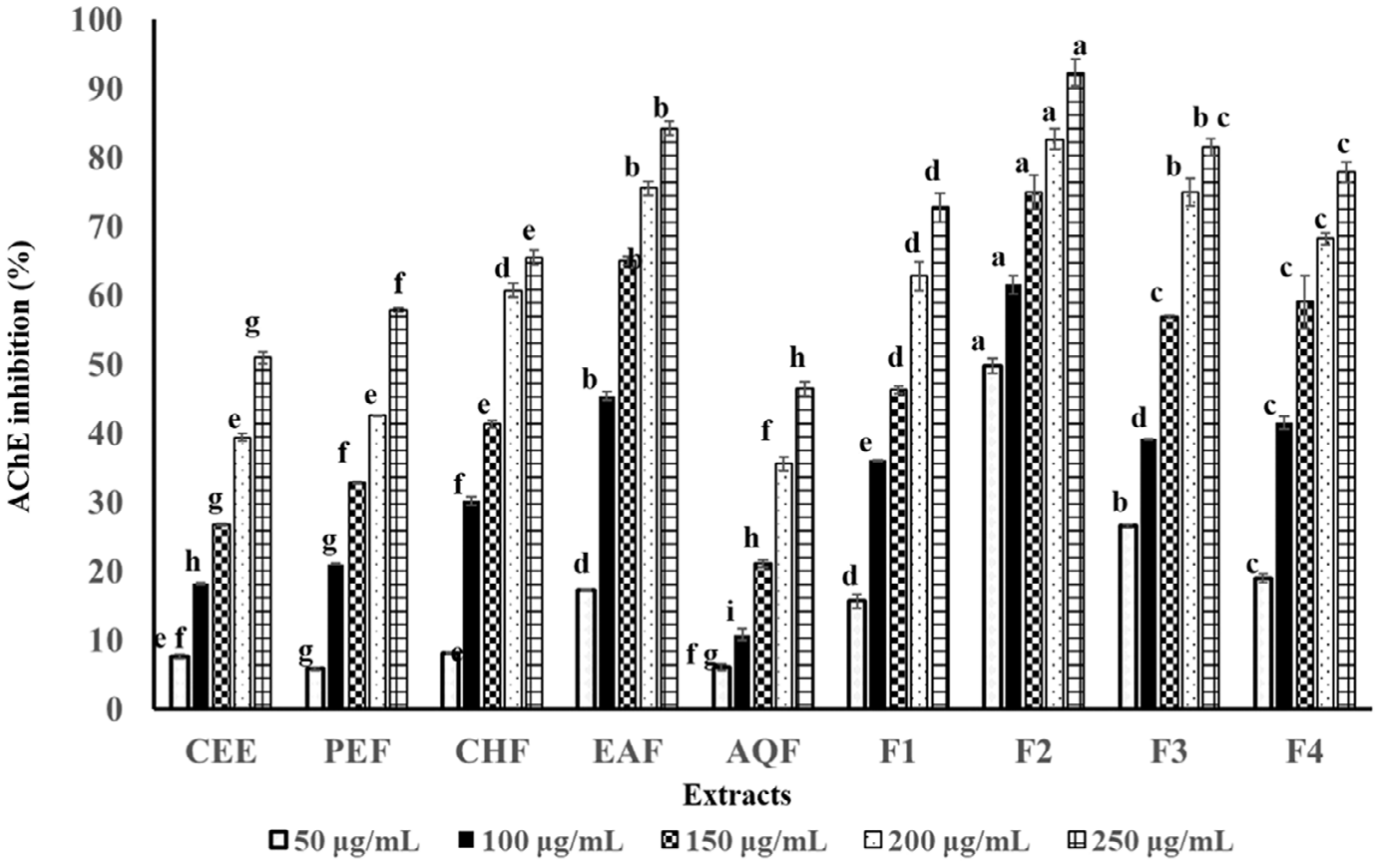
AChE inhibition of different peanut shell extracts. Data are shown as mean ± SD (*n* = 3). Means within the same color (concentration) with different letters significantly differ by Tukey's test at *p* < 0.05.

Peanut shell extract and its fractions were evaluated for AChE inhibition using the Ellman method. All samples exhibited dose‐dependent inhibition, with significant differences in inhibitory activity observed among the tested samples (Figure 4). The inhibition ranged from 5.84% to 49.78% at 50 μg/mL and increased to 46.43%–92.27% at 250 μg/mL. Among the samples tested, fraction F2 showed the strongest inhibitory effect, achieving 92.27% inhibition at 250 μg/mL and an IC_50_ value of 47.22 μg/mL. This was followed by EAF with an IC_50_ value of 107.56 μg/mL, fraction F3 (IC_50_ = 133.64 μg/mL), fraction F4 (IC_50_ = 140.30 μg/mL), fraction F1 (IC_50_ = 148.63 μg/mL), CHF (IC_50_ = 160.22 μg/mL), and PEF (IC_50_ = 232.08 μg/mL). These fractions exhibited enhanced AChE inhibitory activity compared to the CEE, which had an IC_50_ value of 246.82 μg/mL (Table 3).

### 3.5 Correlation Between TFC and Bioactivities of the Extracts and Fractions From Peanut Shells

**FIGURE 5:**
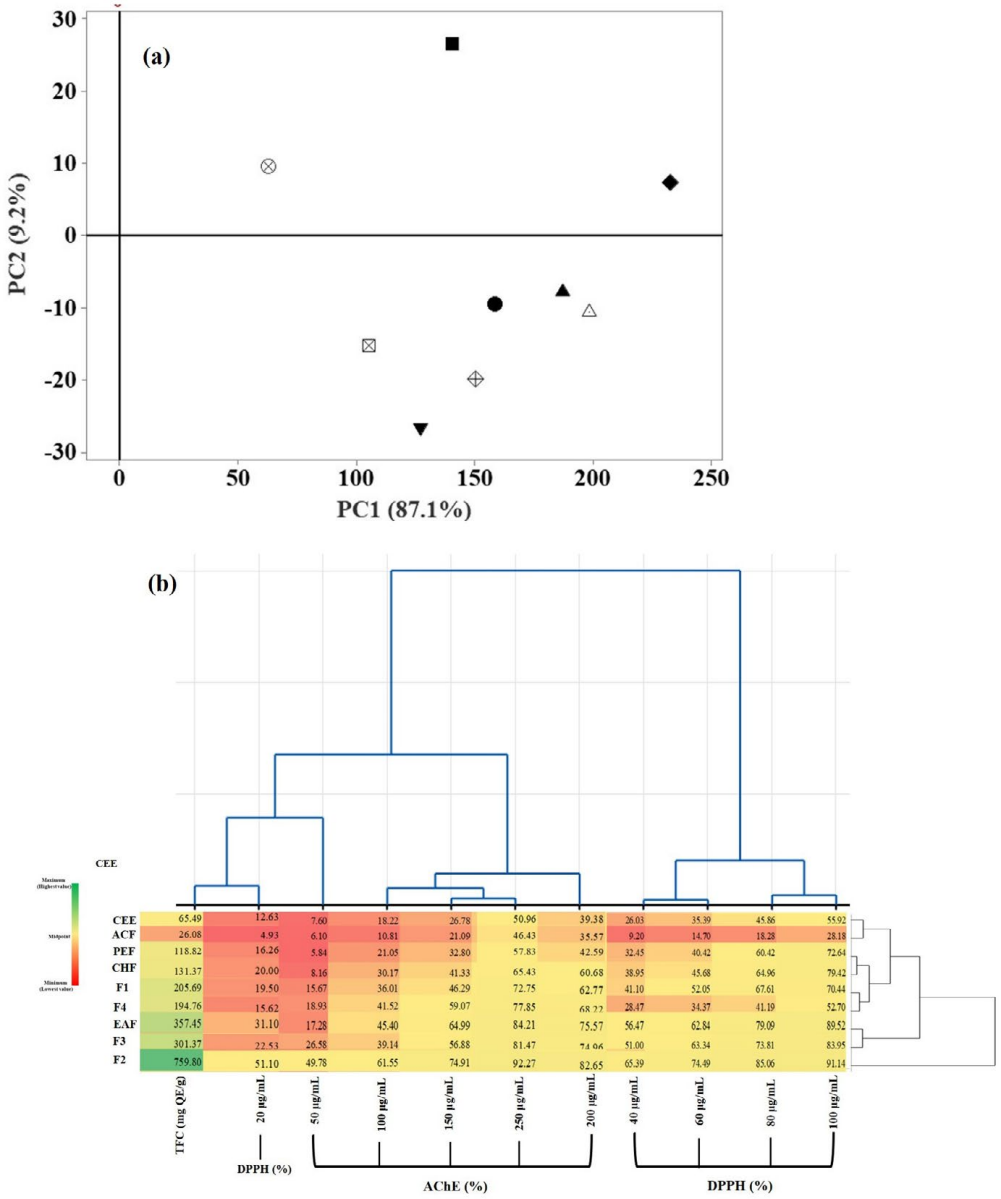
Score plot of PCA (a) and Heatmap clustering analysis (b) of the crude ethanolic extract and its fractions obtained by liquid–liquid partitioning and column chromatography techniques. 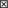, crude ethanolic extract (CEE); 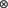, acetone fraction (ACF); 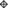, chloroform fraction (CHF); 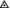, ethyl acetate fraction (EAF); ▼, petroleum ether fraction (PEF); ●, fraction F1; ♦, fraction F2; ▲, fraction F3; and ■, fraction F4.

A multivariate analysis was performed to evaluate the variations in the TFC and bioactive properties of CEE and its eluted fractions obtained from peanut shells. PCA was employed to explore the interactive effects of the extracts and fractions on the bioactive attributes. The PCA results revealed that the first principal component (PC1) accounted for 87.1% of the total variability, with the second component (PC2) contributing an additional 9.2%, together explaining 96.3% of the variance (Figure 5a). The loading plot showed strong positive correlations between TFC, DPPH radical scavenging activity, and AChE inhibition (*R* = 0.979, *p* < 0.05), indicating that TFC significantly contributes to both DPPH scavenging and AChE inhibitory activities.

To further evaluate the effect of fractionation on bioactive properties of peanut shell extracts, a heatmap clustering analysis was performed (Figure 5b). The analysis grouped the samples based on concentration ranges rather than specific extracts or fractions, with variations in color and intensity reflecting differences in data values. Green represented maximum values, while red indicated minimum values. The analysis revealed five distinct clusters. Cluster 1 included CEE and ACF, being characterized by low TFC and bioactivity values. Cluster 2 comprised PEF and CHF, showing moderate improvement in TFC and bioactivities. Cluster 3 consisted of F1 and F4, and Cluster 4 comprised EAF and F3, with midrange values. Cluster 5 (Fraction 2) showed the highest values across all tested parameters (TFC and bioactivities).

Overall, these analyses reveal significant variations in flavonoid and quercetin content and bioactivity among the fractions, demonstrating that liquid–liquid partitioning followed by silica gel column chromatography is an effective strategy for enriching flavonoids and enhancing the antioxidant and AChE inhibitory activities of peanut shell extracts.

## 4 Discussion

The method and solvent employed for extraction and fractionation play an important role in determining the biological activity of flavonoid‐enriched extracts (Munhoz et al. 2014; Liao et al. 2021; Tran et al. 2024). Therefore, identifying optimal extraction and fractionation techniques to enhance bioactive flavonoid yield is required. In this study, liquid–liquid partitioning followed by silica gel column chromatography was used to fractionate the CEE from peanut shells. Through liquid–liquid partitioning, four fractions (EAF, CHF, PEF, and ACF) were obtained. Quantitative analysis revealed that EAF contained the highest TFC, followed by CHF, PEF, CEE, and ACF, with values ranging from 26.08 to 357.45 mg/g (Table 1). These results demonstrated the influence of solvent polarity on extraction efficiency, with acetone proving to be the least effective solvent for flavonoid extraction. Consistent with previous studies, flavonoid extraction efficiency is influenced by physicochemical properties such as hydrophobicity, solubility, and acid–base characteristics of the compounds (Wen et al. 2018; Martínez‐Patiño et al. 2019; Çiftçi and Suna 2022). EtOAc, with its moderate polarity, selectively extracts flavonoids compounds including quercetin, while minimizing the extraction of non‐target materials, yielding flavonoid‐rich extracts from peanut shells (De Rodríguez Luna et al. 2020; Kiene et al. 2023; Sorita et al. 2023).

Given the superior TFC of EAF, silica gel chromatography was employed to further isolate flavonoids from the EAF. This process yielded four fractions (F1–F4), with TFC significantly increasing from 357.45 mg QE/g in EAF to 759.80 mg QE/g in fraction F2. In addition, quercetin and other flavonoids were identified in the CEE and its fractions. TLC analysis revealed 3–6 distinct compounds across the CEE, EAF, and fractions F1–F4. All samples contained a compound with an *R*_f_ value of 0.44, matching the quercetin standard. Dark brown spots observed on the TLC plates were consistent with flavonoid standards (Figure 1, Table 2). Most observed spots had *R*_f_ values below 0.65, indicating the dominance of non‐polar phytochemicals in these extracts (Gomathi et al. 2012; Akhtar et al. 2022).

The presence of quercetin in peanut shells was confirmed by HPLC, with fraction F2 showing the highest concentration (292.38 μg/g), followed by EAF, F3, F1, F4, and CEE (Table 1). The HPLC chromatograms show several unidentified peaks, notably one at a RT of approximately 9.25 min (Figure 5). The identity of this compound was not confirmed in the current study. However, previous studies have reported the presence of fluteolin, eriodictyol, and their glycosidic derivatives in related samples with similar polarity and UV absorption characteristics (Hassan et al. 2022; Imran et al. 2022; Hao et al. 2023; Kiene et al. 2023; Punia Bangar et al. 2023). These compounds are likely to contribute to the biological activities of the extracts. Fractionation substantially enhanced flavonoid and quercetin yields compared to maceration, with TFC ranging from 26.08 to 759.80 mg/g across fractions (F2 > EAF > F3 > F1 > F4 > CHF > PEF > CEE > ACF) (Table 1). The increase in TFC positively correlated with antioxidant properties, as higher TFC enhanced DPPH radical scavenging activity (Table 3, Figure 3). Previous studies have reported that peanut extracts exhibit significant antioxidant properties due to their high flavonoid and phenolic contents, with variations depending on species and extraction methods (Fidrianny et al. 2014; Adhikari et al. 2019; Hassan et al. 2022; Qiu et al. 2012; Sorita et al. 2023). In this study, a hexane:ethyl acetate mixture at a ratio of 70:30 (v/v) was determined as the optimal solvent system for flavonoid extraction by balancing the hydrophobic and hydrophilic properties of flavonoids, resulting in higher yield and purity. Consequently, fraction F2 exhibited the highest DPPH scavenging activity. Reported strong antioxidant activities in fractions obtained from *Origanum* species using chromatographic techniques, including column chromatography and semi‐preparative HPLC. The present study specifically focused on evaluating the antioxidant potential of the flavonoid‐rich extracts and did not assess their potential pro‐oxidant effects. It is important to note that antioxidant activity can vary depending on the redox environment and assay conditions. To gain a more comprehensive understanding of the redox behavior of these compounds, future studies should incorporate complementary antioxidant assays (e.g., ABTS, FRAP, ORAC) and oxidative stress models.

The AChE inhibitory activity of peanut shell extract and its fractions was assessed using the Ellman method, a widely accepted colorimetric assay for screening potential anti‐AD agents (Murray et al. 2013; Ferreira et al. 2020). All samples exhibited dose‐dependent inhibition (IC_50_ = 47.22–246.82 μg/mL), except for ACF (IC_50_ > 250 μg/mL) (Table 3). Fraction F2 showed the strongest inhibition (IC_50_ = 47.22 μg/mL), although it remained moderate compared to the reference standard, berberine chloride. According to potency classifications (Murray et al. 2013; Santos et al. 2018), the extracts demonstrated moderate to low AChE inhibition. Further evaluations using additional reference compounds and in vivo studies are warranted.

Flavonoids, particularly luteolin and quercetin, likely contribute to the observed AChE inhibition. As AChE degrades neurotransmitters, its inhibition could help mitigate cognitive decline, supporting the neuroprotective potential of peanut shell extracts (Han et al. 2022; Sorita et al. 2023). A positive correlation between TFC and AChE inhibitory activity was observed, consistent with previous studies (Ferreira et al. 2020; Taqui et al. 2022). Extraction methods strongly influenced potency, with mid‐polar solvents like EtOAc enhancing flavonoid yields (Eldeen et al. 2005; Chaves et al. 2020; Dean 2020; Kiene et al. 2023; Sorita et al. 2023). Column chromatography further enriched flavonoid content in EAF, leading to more than a twofold increase in both antioxidant and AChE inhibitory activities. To further investigate the changes in flavonoid compositions across various peanut shell extracts, additional studies involving cell‐based assays and animal models are required to confirm the observed in vitro bioactivity in cellular and in vivo systems.

## 5 Conclusions

This study successfully developed an efficient extraction and purification process to enrich bioactive flavonoid compounds from Vietnamese peanut shells. The optimized method, combining ethanol extraction, liquid–liquid partitioning, and silica gel column chromatography, yielded a highly flavonoid‐rich fraction (F2) with a TFC of 759.80 mg QE/g. The resulting fractions demonstrated significantly enhanced bioactivities, particularly in DPPH scavenging and AChE inhibitory activity. Among the fractions, fraction F2 exhibited the most potent activity, with IC_50_ values of 16.00 μg/mL for DPPH scavenging and 47.22 μg/mL for AChE inhibition. These results underscore the potential of peanut shells as an affordable and accessible source of natural antioxidants and AChE inhibitors. Furthermore, the flavonoid‐rich fractions obtained show promise for application in health‐promoting functional foods and nutraceuticals.

## Author Contributions

**Tuyet Nhung Tran:** data curation (equal), formal analysis (equal), investigation (equal). **Quang Tien Ho:** data curation (equal), investigation (equal).**Nhat Tan Nguyen:** investigation (equal), validation (equal). **Thao Vy Nguyen:** methodology (equal), writing – review and editing (equal). **Anh Vo Bui:** validation (equal). **Van‐Son Dang:** data curation (equal). **Dieu‐Hien Truong:** conceptualization (equal), methodology (equal), resources (equal), supervision (equal), writing – original draft (equal). **Hoang Chinh Nguyen:** conceptualization (equal), methodology (equal), supervision (equal), writing – original draft (equal). **Colin J. Barrow:** project administration (equal), supervision (equal), writing – review and editing (equal).

## Ethics Statement

The authors have nothing to report.

## Conflicts of Interest

The authors declare no conflicts of interest.

## Data Availability

All experimental data used to support the findings of this study are completely available from the corresponding author upon request.
